# Bees display limited acclimation capacity for heat tolerance

**DOI:** 10.1242/bio.060179

**Published:** 2024-03-19

**Authors:** Victor H. Gonzalez, Natalie Herbison, Gabriela Robles Perez, Trisha Panganiban, Laura Haefner, Thomas Tscheulin, Theodora Petanidou, John Hranitz

**Affiliations:** ^1^ Undergraduate Biology Program and Department of Ecology and Evolutionary Biology, University of Kansas, Lawrence, KS, 66045, USA; ^2^ Department of Agriculture, University of Puerto Rico, Mayaguez, PR, 00681-9000, USA; ^3^ Department of Biological Sciences, California State University, Los Angeles, CA, 35229, USA; ^4^ Biology Department, Waynesburg University, PA, 47243, USA; ^5^ Laboratory of Biogeography and Ecology, Department of Geography, University of the Aegean, University Hill, Mytilene, 81100, Greece; ^6^ Department of Biology, Commonwealth University of Pennsylvania, Bloomsburg, 17815 PA, USA

**Keywords:** Critical thermal maximum, Climate change, Heat weaves, Heat hardening, Heat priming

## Abstract

Bees are essential pollinators and understanding their ability to cope with extreme temperature changes is crucial for predicting their resilience to climate change, but studies are limited. We measured the response of the critical thermal maximum (CT_Max_) to short-term acclimation in foragers of six bee species from the Greek island of Lesvos, which differ in body size, nesting habit, and level of sociality. We calculated the acclimation response ratio as a metric to assess acclimation capacity and tested whether bees’ acclimation capacity was influenced by body size and/or CT_Max_. We also assessed whether CT_Max_ increases following acute heat exposure simulating a heat wave. Average estimate of CT_Max_ varied among species and increased with body size but did not significantly shift in response to acclimation treatment except in the sweat bee *Lasioglossum malachurum*. Acclimation capacity averaged 9% among species and it was not significantly associated with body size or CT_Max_. Similarly, the average CT_Max_ did not increase following acute heat exposure. These results indicate that bees might have limited capacity to enhance heat tolerance via acclimation or in response to prior heat exposure, rendering them physiologically sensitive to rapid temperature changes during extreme weather events. These findings reinforce the idea that insects, like other ectotherms, generally express weak plasticity in CT_Max_, underscoring the critical role of behavioral thermoregulation for avoidance of extreme temperatures. Conserving and restoring native vegetation can provide bees temporary thermal refuges during extreme weather events.

## Teaser

**Summary:** Bees display limited capacity to increase their heat tolerance following short-term acclimation and acute heat exposure.

## INTRODUCTION

Climate change is considered one of the main drivers of biodiversity loss. Therefore, one of the foremost challenges in contemporary ecology and conservation is to understand the responses of organisms to climate change (e.g. Spooner et al., 2018; Kellermann and van Heerwaarden, 2019; Soroye et al., 2020; Arneth et al., 2020; Harvey et al., 2023). Multiple aspects of the climate are expected to be altered under climate change, including temperature, CO_2_ concentration, UV exposure, rainfall patterns, as well as the intensity, frequency, and duration of extreme weather events, such as cold snaps and heat waves (IPCC, 2021; Meehl and Tebaldi, 2004). The effects of increasingly variable and intense temperatures experienced by organisms during these extreme events are diverse and highly detrimental for most taxa (Thakur et al., 2022). For example, heat waves can be lethal if ambient temperature exceeds organisms’ upper thermal limits but can also lead to behavioral or physiological changes that reduce fitness and organism roles within the ecosystem (e.g. Thakur et al., 2022; Bell et al., 2023; Campion et al., 2023).

Phenotypic plasticity, the ability of an organism to express different phenotypes in response to environmental conditions (West-Eberhard, 2003), has been recognized as one of the most important mechanism for rapid response to climate change (e.g. Matesanz et al., 2010; Bonamour et al., 2019). Plasticity in critical thermal maximum (CT_Max_), the maximum tolerable temperature (e.g. Lutterschmidt and Hutchison, 1997), can enhance organisms’ capacity to withstand or to recover from elevated temperatures. This plasticity can be achieved via thermal acclimation following chronic or brief exposures to thermal stimuli, potentially inducing upregulation of heat-shock proteins or other molecular chaperones that mitigate cellular damage under stress (Angilletta, 2009; González-Tokman et al., 2020). Thus, as thermal plasticity might promote survival and influence patterns of selection, understanding acclimation responses is a key challenge in climate change biology (Kellermann and van Heerwaarden, 2019; González-Tokman et al., 2020; Weaving et al., 2022).

Insects are the most diverse, ecologically significant, and economically vital group of multicellular organisms on the planet, both in freshwater and terrestrial ecosystems (Grimaldi and Engel, 2005). However, they may also be the most vulnerable to climate change due to their limited capacity to regulate body temperature and water loss, given their small body size, high surface area to volume ratio, low fat storage, and relatively high metabolic rate (e.g. Angilletta, 2009; Harrison et al., 2012; Bujan et al., 2016). Consequently, the responses of insects to climate change via thermal plasticity have been an important topic of research and debate, as plastic responses might vary among populations, species, and taxonomic groups, and might also be influenced by both biotic and abiotic factors (Shah et al., 2017; Weaving et al., 2022). For example, species from more thermally variable environments are predicted to have greater capacity to increase CT_Max_ relative to species from thermally homogenous environments, as more variable climates should select for broader thermal niches (‘climate variability hypothesis’, Janzen, 1967; Ghalambor et al., 2006). On the other hand, plasticity in CT_Max_ is expected to be low in species with high CT_Max_ because of the energetic costs involved in maintaining a high heat tolerance (‘trade-off hypothesis’, Somero, 2010; Barley et al., 2021).

Recent systematic reviews indicate that thermal acclimation of critical thermal limits is relatively weak across ectotherms, particularly in insects, but with a more pronounced response in the critical thermal minimum (CT_Min_) when compared to the CT_Max_ (e.g. Gunderson and Stillman, 2015; Gunderson et al., 2017; Barley et al., 2021; Weaving et al., 2022). Thus, thermal plasticity in critical thermal limits might offer limited benefits to insects during extreme climatic events. However, relatively few species have been assessed (∼100 species) for this incredibly diverse group, which raises concerns about whether the limited plasticity in heat tolerance is representative of plasticity for insects in general (Weaving et al., 2022).

Bees are widely recognized as key organisms due to their essential roles in plant reproduction, ecosystem maintenance, and food security (Klein et al., 2007). With over 20,000 species of bees worldwide (Michener, 2007), several studies have documented changes in community composition, population vigor, species distributions, and plant–bee interactions due to landscape-level alterations and climate change (Bartomeus et al., 2013; Kerr et al., 2015; Soroye et al., 2020; Kammerer et al., 2021; Jackson et al., 2022). Nonetheless, information of the thermal biology of bees, including the impact of extreme weather events on their heat tolerance and plasticity remains relatively poorly studied (Gonzalez et al., 2022a,b; Johnson et al., 2023; Quinlan et al., 2023; Sepúlveda and Goulson, 2023).

To improve our understanding of how bees might be impacted by extreme weather events, herein we measured the short-term acclimation ability of the CT_Max_ in foragers of six bee species from the Greek island of Lesvos. In addition, we assessed whether CT_Max_ increases following acute heat exposure simulating a heat wave. Bees occupy diverse thermal environments and vary dramatically in morphology and life history traits (Michener, 2007), which suggests potential differential responses to heat stress. Thus, we assessed species that differed in body size, nesting habit, and level of sociality, ranging from very small, social, and ground-nesting species [*Lasioglossum malachurum* (Kirby)] to large, solitary, and wood-nesting species [*Xylocopa violacea* (Linnaeus)]. Body size determines foraging distance (Greenleaf et al., 2007) and thus large bees are likely to experience a greater range of temperatures during their daily foraging trips than do small bees. Therefore, we hypothesized that larger bees would display greater plasticity in CT_Max_ when compared with small bees, an expectation consistent with the climatic variability hypothesis (Janzen, 1967; Ghalambor et al., 2006). In addition, following the trade-off hypothesis (Somero, 2010), we predicted a negative relationship between plasticity in CT_Max_ and innate CT_Max_ among bee species.

## RESULTS

### Acclimation capacity

After accounting for body size, we found significant differences in CT_Max_ among species (ANCOVA, Wald χ^2^=50.87, DF=5, *P*<0.001) but not in the average estimate of CT_Max_ of bees exposed to the heat treatment (Wald χ^2^=2.46, DF=1, *P*=0.12). The interaction between species and heat treatment was not significant (Wald χ^2^=5.08, DF=5, *P*=0.41).

**Fig. 1.**
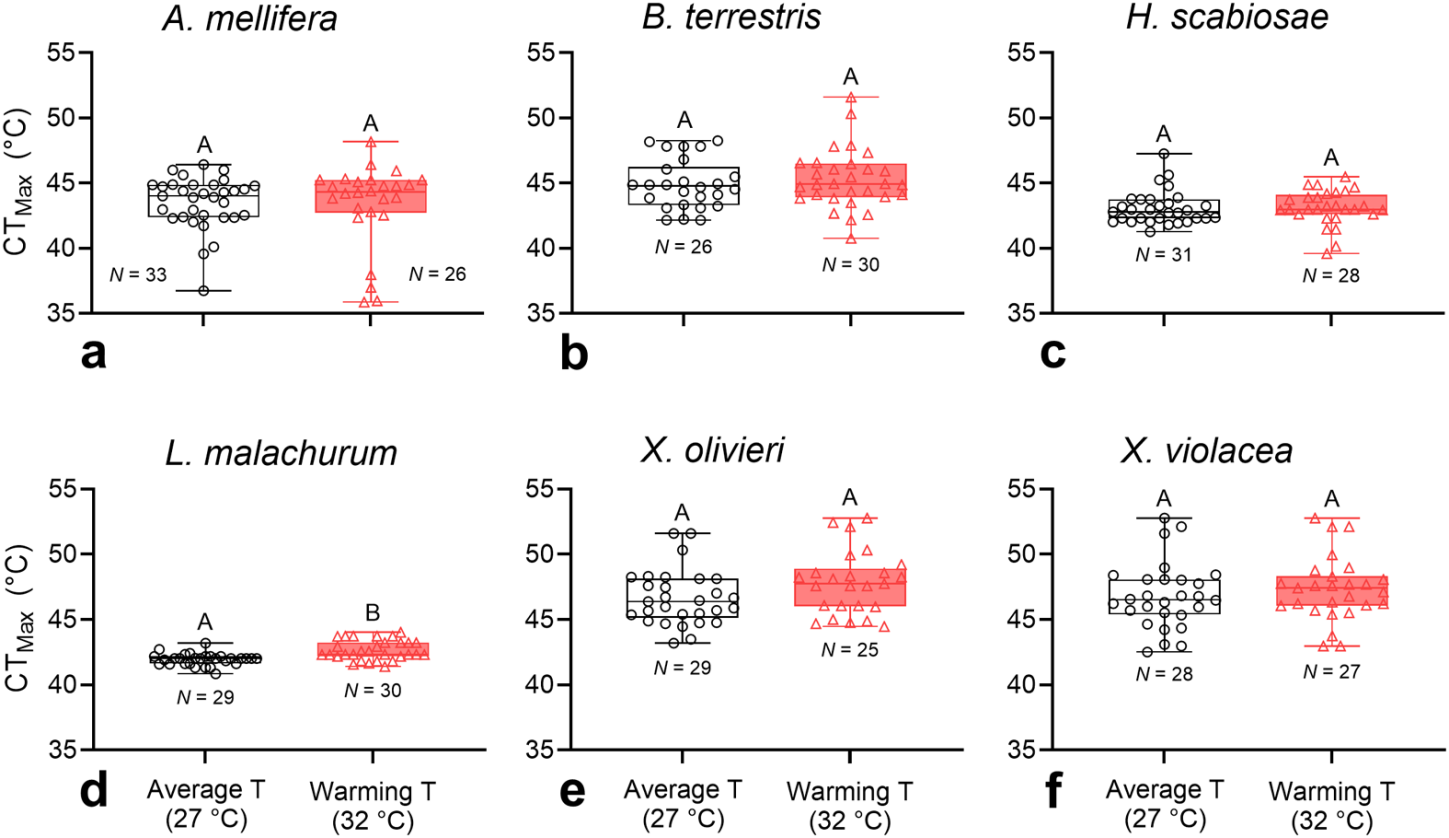
**Boxplots showing critical thermal maxima (CT_Max_) of bee foragers from six species acclimated for 48 h at either 27°C (average ambient temperature) or 32°C (warming temperature).** To better understand the results, we conducted *post-hoc* ANCOVA tests for each species individually. Boxplots display median, quartiles, and extreme values. For each species, different letters above boxplots indicate significant mean differences in the *post-hoc* tests (*P*<0.05).

We conducted an ANCOVA test for each species individually to better understand this result. We found no significant differences between treatments for each species, except for *L. malachurum* (DF=1, *P*<0.001; Fig. 1D, Table S1), which after acclimation had a CT_Max_, on average, 0.72°C greater than that of the control group. Across all species, variance in CT_Max_ was similar between treatments [*F*_(175, 165)_=0.75, *P*=0.06]. Within species, variance of CT_Max_ was also similar between treatments, except for an increase in the CT_Max_ variance of *Apis mellifera* Linnaeus and *L. malachurum* (Table S2).

**Fig. 2.**
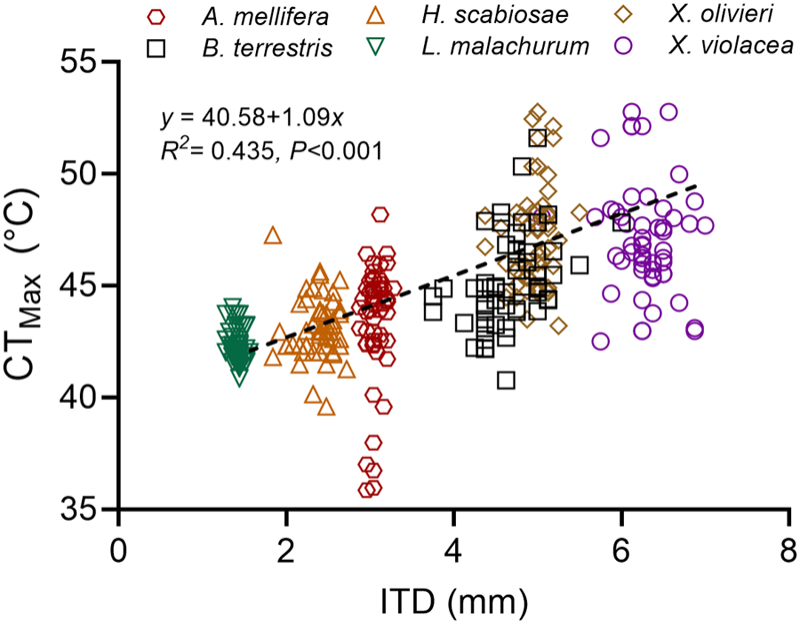
**Relationship between intertegular distance (ITD) and critical thermal maxima (CT_Max_) across all six species of bees assessed in this study.** CT_Max_ values are for bees kept at average ambient temperature (27°C) without prior acclimation.

Bees’ intertegular distance (ITD) in our study ranged from 1.42 mm in *L. malachurum* to 6.29 mm in *X. violacea*, while average estimates of CT_Max_ ranged from 42°C in *L. malachurum* to 47°C in *Xylocopa olivieri* Lepeletier and *X. violacea*. Across all species, CT_Max_ increased with increasing ITD (*P*<0.001, *R*^2^=0.435±0.07; Fig. 2). Within species, CT_Max_ did not significantly increase with increasing ITD, except in *Bombus terrestris* (Linnaeus) (*P*=0.001, *R*^2^=0.163±0.65; Table S3). Except for *A. mellifera*, the acclimation response ratio (ARR) was positive for each species, demonstrating the ability to increase CT_Max_ in response to acclimation. For every 1°C rise in acclimation temperature, ARR for CT_Max_ ranged from as low as 0.008°C in *Halictus scabiosae* (Rossi) to as high as 0.234°C in *X. olivieri*. However, ARR was not significantly associated with bees’ ITD (*P*=0.548, *R^2^*=−0.129±0.03; Fig. S2) or with CT_Max_ (*P*=0.356, *R^2^*=0.017±0.02; Fig. S3).

### Response to acute heat exposure

**Fig. 3.**
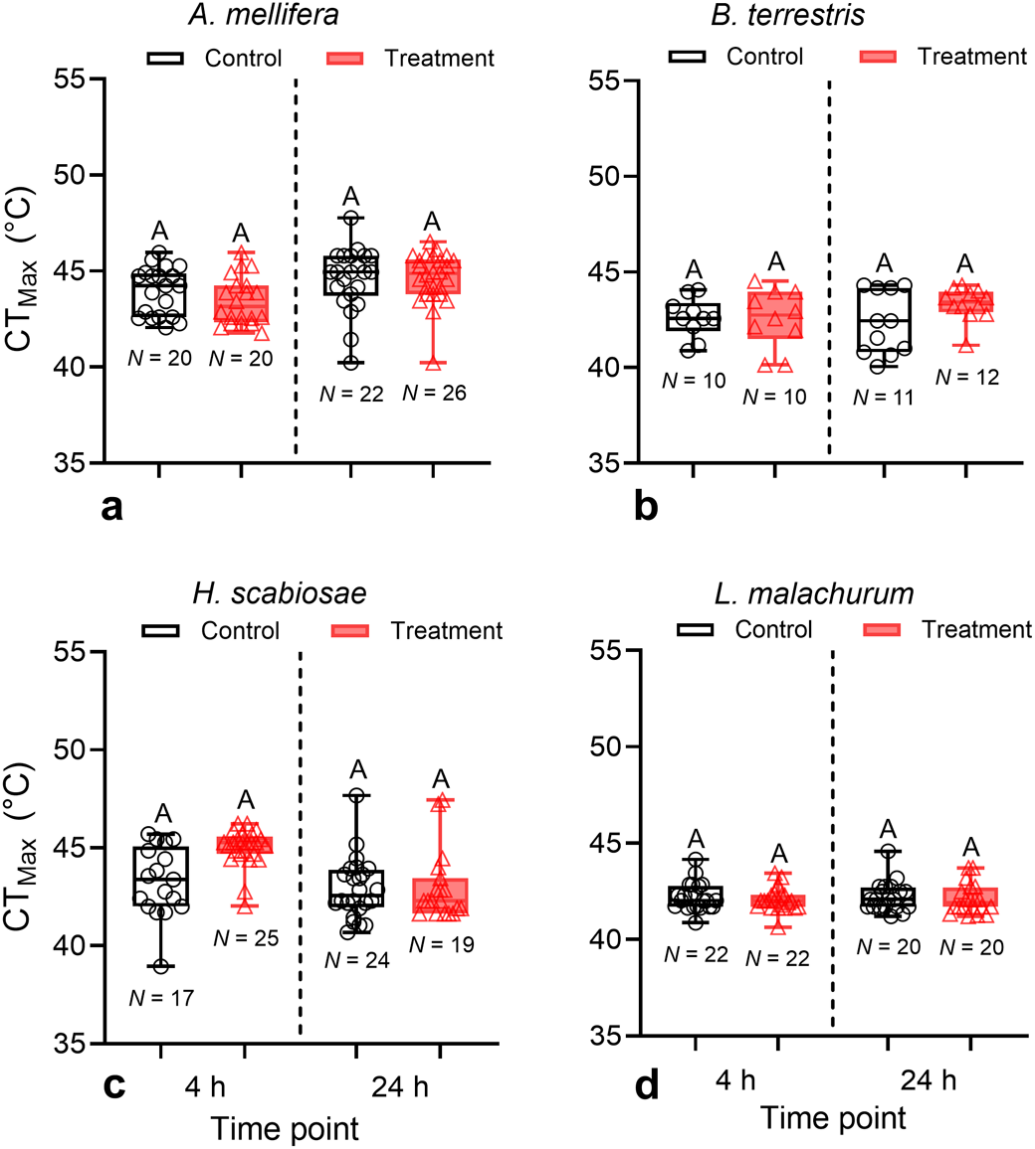
**Boxplots showing critical thermal maxima (CT_Max_) of bees following a 4-h acute heat exposure (38°C) and 24 h after heat exposure (treatment).** As controls, bees were kept at 27°C. Boxplots display median, quartiles, and extreme values. For each figure, different letters above boxplots indicate significant mean differences (*P*<0.05).

After accounting for body size, CT_Max_ differed significantly among species (Wald χ^2^=28.97, DF=3, *P*<0.001), but not between treatments (Wald χ^2^=0.01, DF=1, *P*=0.93) or in the time following an acute heat exposure (Wald χ^2^=0.01, DF=1, *P*=0.91). All interactions among factors were not significant (Fig. 3A-D, Tables S4, S5). Variance in CT_Max_ was similar between treatments [*F*_(145, 153)_=0.99, *P*=0.93] and timing [*F*_(153, 145)_=1.19, *P*=0.30].

## DISCUSSION

Our results demonstrate that bees in our study have limited capacity to increase CT_Max_ following either short-term acclimation to warm temperatures or acute heat exposure. The average estimate of CT_Max_ did not significantly increase when bees were acclimated for 48 h to a temperature 5°C higher than the average ambient temperature. Across all species, variance in CT_Max_ was not impacted by the heat treatment, although it increased the variance of *A. mellifera* and *L. malachurum* (Table S2). Thus, these results support the idea that plasticity in CT_Max_ is relatively weak in insects (Weaving et al., 2022).

It is possible that the acclimation temperature we chose (32°C), as well as the incubation period (48 h), may have constrained bees’ ability to increase their CT_Max_. It is also possible that bees were already acclimated to the warm summer temperatures at the time of the study. However, Oyen and Dillon (2018) and Sepúlveda and Goulson (2023) used comparable or higher (36°C) temperatures in their 72-h acclimation assays for the North American bumble bee *B. impatiens* Cresson and the European bumble bee *B. terrestris*. In both cases, the average CT_Max_ did not improve with acclimation temperature. Thus, the observed limited acclimation capacity to heat tolerance in our study may be a general pattern among bees, and not a methodological artifact. However, we assessed a reduced number of species from a single location and *post-hoc* tests on individual species revealed an effect of the acclimation temperature in *L. malachurum* (Fig. 1D). Future studies should assess a larger number of bee species from several populations, as well as their responses to both constant and fluctuating acclimation temperatures. While studies often assess acclimation capacity using constant temperatures in the laboratory, organisms may respond differently to fluctuating acclimation temperatures, which better reflect the thermally variable natural environment that they experience (e.g. Sgrò et al., 2016). To date, thermal bee studies have only used constant acclimation temperatures.

The weak plasticity in CT_Max_ displayed among bees is reflected in the small values of ARR we calculated from the acclimation assays (Table 1). On average, for every 1°C rise in acclimation temperature, bees’ CT_Max_ increased by 0.09°C, which is comparable to the average ARR calculated for all insects in the recent meta-analysis by Weaving et al. (2022). The negative ARR value displayed by *A. mellifera* (–0.028) indicates the opposite trend observed for all other bees, that is, a reduction in the CT_Max_ with an increase in the acclimation temperature. At our study sites, mean hourly air temperature ranges from 16°C to 38°C in the shade (Gonzalez et al., 2020), but bees may experience much higher temperatures when foraging under the sun. For example, in foragers of bumble bees and carpenter bees, body temperature can be as high as to 2–3°C lower than their CT_Max_, demonstrating that bees forage at or near their thermal limits (Gonzalez et al., 2020; Naumchik and Youngsteadt, 2023). Heat waves pose an even greater risk of overheating for foraging bees, as ambient temperatures can rise well above 40°C for several consecutive days [Copernicus Climate Change Service (C3S), 2023]. Thus, our results suggest that bees not only might have an overall weak plasticity in their CT_Max_ to cope with these changes in temperature but also potential performance declines, although the latter could have resulted from the stress of holding bees in vials during the experiment.

**Table 1.**
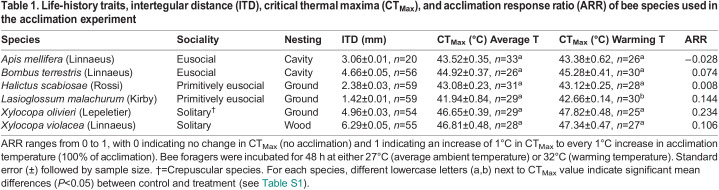
Life-history traits, intertegular distance (ITD), critical thermal maxima (CT_Max_), and acclimation response ratio (ARR) of bee species used in the acclimation experiment

We predicted that large bees would display greater plasticity in CT_Max_ when compared with small bees and that species with high CT_Max_ will display low plasticity in their CT_Max_. Contrary to our expectations, we found no association between ARR and body size or CT_Max_. Thus, our observations do not support these predictions based on the climatic variability hypothesis or the trade-off hypothesis, but we only assessed a reduced number of species. Nonetheless, support for two these hypotheses is mixed in the literature, with some studies favoring either hypothesis. Such a lack of consensus among studies might be due to the lack of broad-scale comparative empirical studies, the lack of a phylogenetic framework to account for potential phylogenetic effects, or by not considering the effect of behavior in regulating species exposure to the environment (Gunderson and Stillman, 2015; Kingsolver and Buckley, 2017; Kellermann et al., 2018). Future studies should test these hypotheses using a higher number of species at a greater spatial scale.

Our findings revealed a significant increase in CT_Max_ across all species as ITD increased (Fig. 2). This indicates that large-bodied species, such as the bumble bee *B. terrestris* and the carpenter bees *X. olivieri* and *X. violacea*, can tolerate higher temperatures than small-bodied species like *L. malachurum*. Such a positive relationship between heat tolerance and body size is expected, given that small bees heat up more quickly than large bees due to their high surface area to volume ratio, which increases convective heat transfer (Johnson et al., 2023; Oyen et al., 2016; Gonzalez et al., 2022c). However, such a relationship between CT_Max_ and body size is not always straightforward, as it seems to vary among bee communities, lineages, and species. For example, while some studies provide support for this relationship (Oyen et al., 2016; Gonzalez et al., 2022c), others find no effect of body size on this thermal trait (Hamblin et al., 2017; Oyen and Dillon, 2018; Gonzalez et al., 2020; 2022a; Jones et al., 2024). Similarly, the influence of body size on CT_Max_ appears to be species specific, even varying within species of the same genus as in the case of bumble bees (Oyen et al., 2016; Oyen and Dillon, 2018; Gonzalez et al., 2022a). Unlike the study by Sepúlveda and Goulson (2023), where CT_Max_ of *B. terrestris* remained unaffected by body size, we found that *B. terrestris* was the only species in which CT_Max_ increased with increasing ITD. Considering that several distinct populations or subspecies of *B. terrestris* have been recognized across its distributional range (Rasmont et al., 2008), our work indicates varied responses in this thermal trait at the population level.

Our study also revealed that bees’ average CT_Max_ did not increase following acute heat exposure or 24 h after the exposure (Fig. 3). These results suggest that the prior heat exposure in our experiments did not prepare bees to tolerate further heat exposure. An improvement of heat tolerance due to the upregulation of heat shock proteins has been documented in other insects (González-Tokman et al., 2020), and honey bees and bumble bees are known to upregulate these proteins when exposed to thermal stress (Koo et al., 2015; Pimsler et al., 2020; Al-Ghzawi et al., 2022). While unanticipated, our results agree with those of a recent study by Quinlan et al. (2023) in which *B. impatiens* did not show improvement on its heat tolerance, as measured as the time to heat stupor, after brief or longer exposures to heat stress. It is likely that inducible heat shock proteins are variable in their expression within bee species and populations (González-Tokman et al., 2020). If climate change may result in bees operating more closely to their upper thermal limits, it will be important to know how heat-shock proteins could mediate local adaptations to heat. Unfortunately, this aspect remains largely unexplored in bees.

CT_Max_ has been assessed in a relatively small number of bee species, most of them from North America (Gonzalez et al., 2022a,b; Johnson et al., 2023). However, our estimates of CT_Max_ fall within the range reported for other species, with the highest values recorded for bumble bees and carpenter bees. Except for *L. malachurum* and *H. scabiosae*, CT_Max_ has previously been assessed in the remaining species we studied. Maebe et al. (2021) and Sepúlveda and Goulson (2023) estimated CT_Max_ in lab-reared subspecies of *B. terrestris*, and several authors (e.g. Burdine and McCluney, 2019; Sánchez-Echeverría et al., 2019; Gonzalez et al., 2022b; Benito et al., 2024) have assessed various subspecies as well as populations of *A. mellifera*, including feral and managed Africanized honey bees. Gonzalez et al. (2020) assessed CT_Max_ for *X. violacea* and *X. olivieri*, both from the island of Lesvos. There are differences in the average estimates of CT_Max_ between those studies and the values we obtained, which are likely due to methodological variations that include the rate of temperature change in dynamic protocols, the type of equipment used, and the origin of bees (lab-reared versus wild individuals). See Gonzalez et al. (2022a,b) and references therein for a discussion.

### Implications for conservation and future studies

Our results have significant implications for understanding bees’ heat tolerance mechanisms and their potential responses to climate change. The weak plasticity in bees’ CT_Max_ after short-term acclimation, as well as their inadequate ability to improve heat tolerance after prior acute heat exposure, suggest physiological limitations in forager bees to cope with increasingly variable and intense temperatures during extreme climatic events. Consequently, other mechanisms, such as behavioral thermoregulation or nest selection, might be even more important for foraging bees to cope with these extreme climatic events. Indeed, bees can restrict the range of temperatures they experience by adjusting their foraging and nesting activities. For example, bees are known to shorten or shift their foraging schedule (Willmer and Stone, 2004; de Farias-Silva and Freitas, 2021), reduce the number or duration of pollen-foraging trips (Maia-Silva et al., 2015), or regurgitate nectar or water for evaporative cooling (Portman et al., 2021). In addition, social insects are expected to display greater behavioral plasticity to tolerate environmental changes when compared to solitary species (Parr and Bishop, 2022). Social species are not only able to thermoregulate their nest (Jones and Oldroyd, 2007), but they may also display within-group variation in the thermal tolerance across colony members, as documented in ants (Baudier and O'Donnell, 2017). For solitary species or species unable to thermoregulate the nests, optimal nest selection or structural adjustments to their nests are critical to influence the thermal environment they experience (Richards et al., 2020; Gonzalez et al., 2023). For example, among our focal species, *H. scabiosae* ceases foraging at the heat of the day and seals its nest entrance, which suggests heat-avoidant behavior. However, at least for an Austrian population, such behavior appears to be more related to an increase of nest-infecting parasites than in response to elevated ambient temperatures (Lienhard et al., 2010). The limited physiological plasticity of foraging bees also underlines the significance of conserving and restoring native vegetation to provide them with temporary thermal refuges.

To obtain a more comprehensive understanding of bees’ responses to climate change, future studies should not only attempt to evaluate the acclimation capacity of other bee species at various locations, but also investigate the thermal tolerance and acclimation potential of their immature stages. While adult bees can employ behavioral thermoregulation to cope with temperature fluctuations, this ability is absent in their immobile immature stages. However, greater thermal tolerance and plasticity might be anticipated in these developmental stages compared to adults due to the limited opportunities for behavioral thermoregulation, a prediction consistent with the observed pattern known as the Bogert effect (Gunderson and Stillman, 2015). This could be the case for immature stages of solitary bee species or thermoconform social species, such as stingless bees. A recent study shows that stingless bees' larvae and pupae are indeed more heat tolerant than foragers (Nacko et al., 2023), which agrees with our prediction.

## MATERIALS AND METHODS

### Study site and species

We captured bees for this study during June and July of 2023 at two locations along Kalloni Bay on the Greek island of Lesvos: a coastal semi-natural low scrub (phrygana) habitat in Achladeri, near Ancient Pyrrha (39°09′17.40′′N, 26°16′32.74′′E, 1.3 ma.s.l.) and an unmanaged pasture near Skala Kallonis (39°12′36′′N, 26°12′12′′E, 1.0 ma.s.l.), approximately 9 km northwest of Achladeri. Except for honey bees, which we captured from two experimental Langstroth hives each trained to scented feeders, we collected bees at flowers between 6:00 and 11:00 h with a net and transferred them individually to plastic vials, which we then capped with fabric (with holes ∼1 mm in diameter). We kept bees inside a cooler with an ice pack covered in a piece of paper towel (16–19°C) until we completed fieldwork. We selected the following six species based on their local abundance and differences in life history traits, which included ground and wood nesting habits, solitary and social behaviors, diurnal and crepuscular activity, and varying sizes (Table 1): *Apis mellifera*, *Bombus terrestris*, *Halictus scabiosae*, *Lasioglossum malachurum*, *Xylocopa violacea*, and *X. olivieri*. The senior author identified these species by comparing specimens with the reference bee collection of the Melissotheque of the Aegean at the University of the Aegean, Mytilene, Lesvos, Greece. We attempted to capture at least 30 individuals per species per treatment, but because most bees were collected opportunistically, sample sizes varied among species and treatments. We brought bees to the laboratory and fed them *ad libitum* with a small piece of commercial honey bee food paste (Viepol, Kirka^®^, Greece) placed on a moistened cotton ball with a 50% sucrose solution. Then, we transferred bees to a 25 L reptile incubator (Vevor^®^) where we used them for either the acclimation or acute heat exposure assays, as described below (Fig. S1). Bees were transferred to incubators within 1–2 h after being captured in the field.

### Acclimation assays

We acclimated bees for 48 h in the dark at one of two constant temperature treatments, ‘average’ and ‘warming’. We used 27°C as the average temperature, which represents the mean ambient daily temperature recorded at both study locations the previous year during the summer months. At each site, we used an iButton data logger (DS1923 Hygrochron; Maxim Integrated, San Jose, CA, USA), which we protected from solar radiation with aluminum foil and hung 1 m above ground from tree branches (see Gonzalez et al., 2020). We recorded temperature and humidity every 30 min for seven consecutive weeks from mid-June to late July. We used 32°C as the warming temperature. As in Shah et al. (2017), we used a 5°C increase from the average temperature as the warming temperature, which is within the range of natural variation recorded at the location sites. We conducted experiments in the dark to minimize the effect of potential physiological and behavioral stressors associated with light. We chose the acclimation period of 48 h because pilot studies indicated higher mortality at longer incubation periods. We replenished food every 24 h and kept humidity inside the incubators between 40–60% by adding water to the bottom tray. We used a different set of bees for each acclimation temperature treatment and measured their CT_Max_ as indicated below.

### Acute heat exposure assays

To assess the impact of acute heat exposure on CT_Max_, we placed bees in an incubator at 38°C (40–60% RH) for a 4-h period. We measured CT_Max_ following the acute heat exposure and assessed post-heat exposure recovery after 24 h in an incubator at 27°C. Bees incubated at 27°C for 4 and 24 h served as control groups. For this experiment, we focused on the social species, as individuals of both solitary carpenter bees (*Xylocopa olivieri* and *X. violacea*) became scarcer and difficult to capture towards the end of our field season at the study site. We chose 38°C, which is 11°C higher than the average ambient temperature at our study locations, and an exposure period of 4 h for several reasons. First, this temperature is, on average, about 3°C lower than the CT_Max_ of *L. malachurum*, the species in our study with the lowest CT_Max_. Second, even large bees, such as some bumble bees, may reach heat stupor and eventually die when exposed to 40°C in an incubator for periods as short as 30 min (Martinet et al., 2015). Third, 4-h acute exposures are ecologically relevant simulation of afternoon heat stress that increases *Hsc70* expression in life stages of insects (Feder et al., 1997; Barthell et al., 2002; Hranitz et al., 2009). Blasco-Lavilla et al. (2021) showed significant expression of heat shock genes *Hsc70* in *B. terrestris*, a species with a high CT_Max_ (as shown in Table 1), between 2 h and 6 h after exposure to 38°C. Similarly, some subspecies of the European honey bees are known to upregulate heat shock proteins after 4 h of exposure to 40°C or 45°C (Alqarni et al., 2019). In addition, some solitary bee species, such as leaf cutter bees, expressed heat shock proteins even when exposed to 35°C for 3 h (Barthell et al., 2002). Fourth, studies demonstrate that foraging in at least some bumble bees, including *B. terrestris*, is significantly reduced at temperatures higher than 32°C (e.g. Kwon and Saeed, 2003; Kenna et al., 2021; Hemberger et al., 2023). Thus, we considered 38°C to be a moderately high temperature that would allow us to compare all species, and 4 h treatment as a long enough period for bees to potentially display changes in the expression of heat shock proteins while reducing mortality.

### CT_Max_ assays

We measured bees’ CT_Max_ using a dynamic (ramping temperature) protocol with the Elara 2.0 (IoTherm, Laramie, WY, USA). We placed bees individually inside glass shell vials (either 9×30 mm, 0.92 cm^3^ for small bees or 12×35 mm, 1.80 cm^3^ for large bees) and plugged them with a moistened cotton ball to ensure consistent humidity during the assays. We used an initial temperature of 27°C and held bees for 10 min at this temperature before increasing it at a rate of 0.5°C min^−1^. We chose this rate of temperature change to reduce the time required for each assay and to minimize the effect of confounding physiological stressors, such as dehydration or starvation (Gonzalez et al., 2022b). We placed vials horizontally on the stage to prevent bees from climbing the sides of the vial. To estimate the temperature inside the vials, we placed a K-type thermocouple inside two empty glass vials plugged with a cotton ball. We individually tracked these vial temperatures using a TC-08 thermocouple data logger (Pico Technology, Tyler, TX, USA). CT_Max_ was taken as the temperature at which bees lost muscular control, spontaneously flipping over onto their dorsa and spasming (Lutterschmidt and Hutchison, 1997; García-Robledo et al., 2016, 2018). See Gonzalez et al. (2022b) for details of the thermal apparatus and setting used. Once experiments concluded, we euthanized bees and estimated their body size as indicated below.

### Body size

We estimated body size by measuring the minimum distance between the tegulae (ITD) of each specimen. However, for honey bees, which display little variation in body size, we used an average taken from 20 individuals. ITD is a robust estimator of bee dry mass (Cane, 1987) and it is commonly used in ecological studies as a proxy for body size (Kendall et al., 2019). We measured bees using an ocular micrometer on S6E stereomicroscope (Leica Microsystems, Wetzlar, Germany). Voucher specimens have been deposited in the Melissotheque of the Aegean.

### Statistical analyses

We conducted statistical analyses in R (R Core Team, 2018). We used an ANCOVA test to compare CT_Max_ between bees exposed to average and warming temperature scenarios while controlling for the effects of body size. We implemented a linear mixed-effect model (LMM) using the lmer function in the lme4 package (Bates et al., 2015) with species and treatment (average and warming temperatures) as fixed factors, date of collection as a random factor, and ITD as a covariate. To evaluate the relationship between CT_Max_ and ITD among and within species, we implemented a linear regression analysis using the lm function. To assess the level of acclimation among species, we calculated the acclimation response ratio (ARR) as Δ CT_Max_/Δ Acclimation (Claussen, 1977), where Δ CT_Max_ is the average CT_Max_ after acclimation to the warming temperature (32°C) minus the average CT_Max_ after acclimation to the average temperature (27°C) and Δ acclimation is the difference between the warming and average acclimation temperatures (5°C). This ARR ranges from 0 to 1, with 0 indicating no change in CT_Max_ (no acclimation) and 1 indicating an increase of 1°C in CT_Max_ to every 1°C increase in acclimation temperature (100% of acclimation). To assess whether bees’ acclimation capacity is associated with their body size or CT_Max_, we used a linear regression analysis to assess the relationship between ARR and bees’ average ITD and CT_Max_. For this analysis, we used the average CT_Max_ estimated for bees kept at average ambient temperature, without prior acclimation. To assess changes in CT_Max_ after exposure to heat stress, while controlling for the effects of body size, we used a mixed-model ANCOVA with species, heat treatment (27°C and 38°C) and timing (4 h and 24 h) as fixed factors, date of collection as a random factor, and ITD as a covariate. We assessed the significance of fixed effects using a Type II Wald χ^2^ test with the car package (Fox and Weisberg, 2019). When factors and factor interactions were significant, we used the lsmeans package (Lenth, 2016) to conduct multiple pairwise comparisons with Bonferroni adjustment to assess for differences among groups. We compared variance in CT_Max_ using *F*-tests with the var.test function from the car package.

## Supplementary Material

10.1242/biolopen.060179_sup1 Supplementary information
