# Supplementary material for: Bees display limited acclimation capacity for heat tolerance
Source: Biol Open. 2024 Mar 19;13(3):bio060179. doi: 10.1242/bio.060179 (PMC10979511; doi:10.1242/bio.060179)
Supplement: Supplementary information [file biolopen-13-060179-s1.pdf]

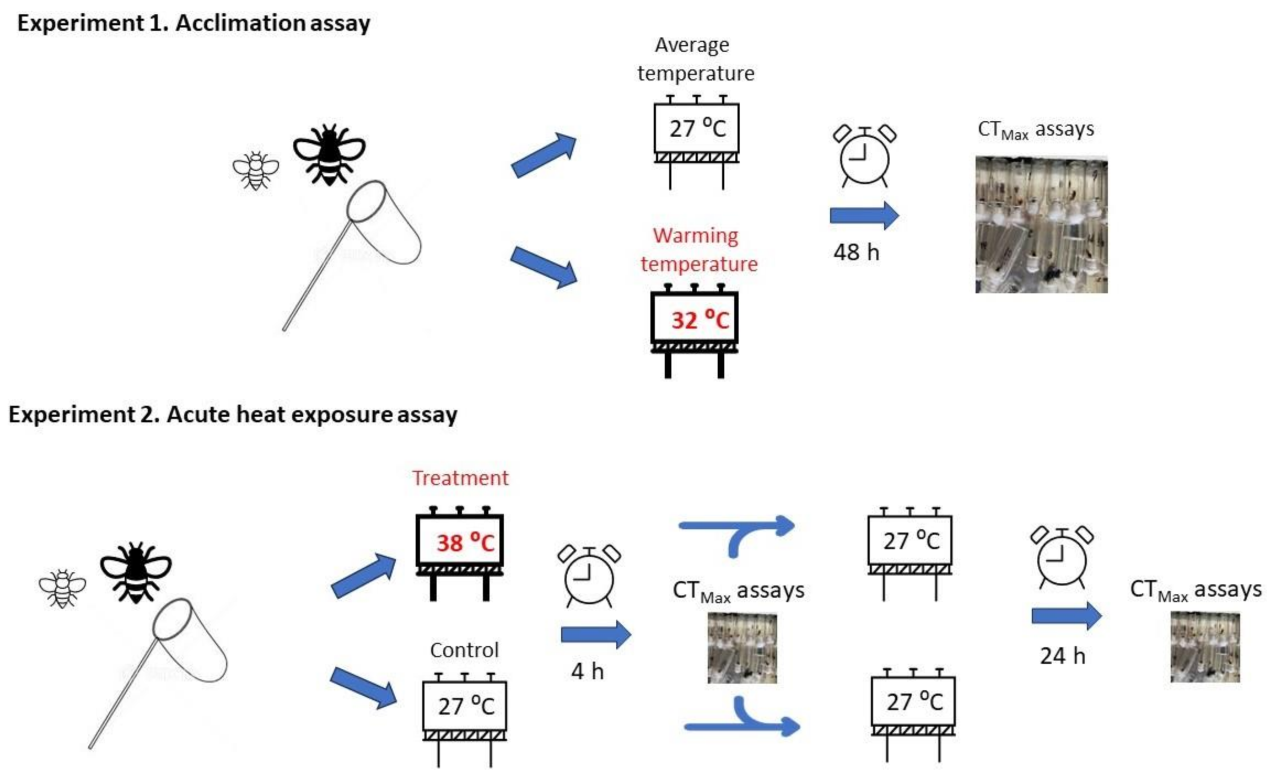

Fig. S1. Graphic summary of the two experiments conducted in this work to assess the effect of acclimation and acute exposure to elevated temperatures on bees' heat tolerance. The identity of species and sample sizes varied among experiments and treatments because most bees were collected opportunistically in the field.

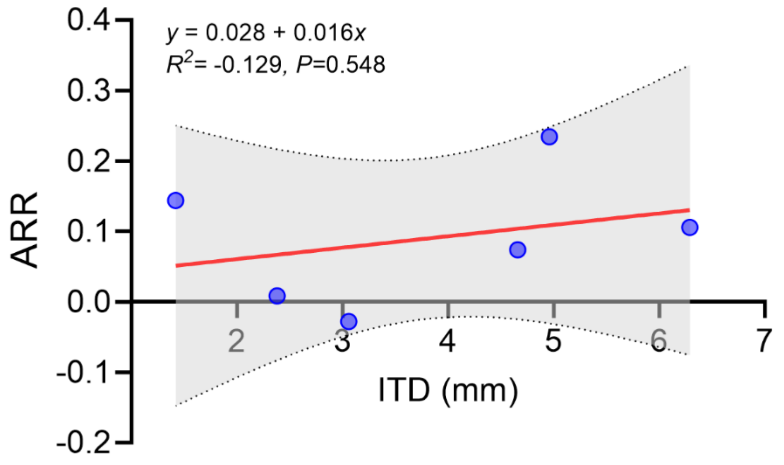

Fig. S2. Relationship between intertegular distance (ITD) and acclimation response ratio (ARR). The trend line represents a linear regression and the grey areas around the line are 95% confidence intervals.

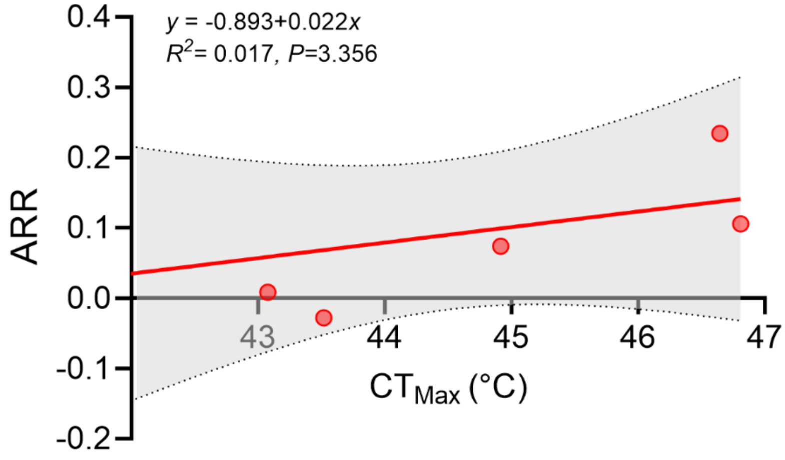

Fig. S3. Relationship between critical thermal maxima (CT) and acclimation response ratio (ARR). The trend line represents a linear regression and the grey areas around the line are 95% confidence intervals.

Biology Open • Supplementary information

Table S1. Results from mixed-model ANCOVA test for each species used in the acclimation experiment to compare average CT_{Max} between bees incubated for 48 hours at either 27 °C (average ambient temperature) or 32 °C (warming temperature). Statistically significant *P*-value in bold face. DF = 1 in all cases.

Species	X^2	*P*-value
Apis mellifera	0.15	0.70
Bombus terrestris	0.54	0.46
Halictus scabiosae	0.04	0.89
Lasioglossum malachurum	17.43	**<0.001**
Xylocopa olivieri	2.53	0.11
Xylocopa violacea	0.44	0.51

Table S2. Results from *F*-tests for each species used in the acclimation experiment to compare variance in CT_{Max} between bees incubated for 48 hours at either 27 °C (average ambient temperature) or 32 °C (warming temperature). Statistically significant *P*-value in bold face.

Species	Variance Average T	Variance Warming T	F	DF	*P*-value
Apis mellifera	4.04	10.0	0.40	32, 25	**0.02**
Bombus terrestris	3.60	5.10	0.71	25, 29	0.38
Halictus scabiosae	1.61	1.81	0.89	30, 27	0.76
Lasioglossum malachurum	0.21	0.56	0.37	28, 29	**0.01**
Xylocopa olivieri	4.46	5.78	0.77	28, 24	0.51
Xylocopa violacea	6.43	6.02	1.07	27, 26	0.87

Biology Open • Supplementary information

Table S3. Results from linear regression analyses which explore the relationship between critical thermal maximum (CT_{Max}) and intertegular distance (ITD) for each bee species used in our study. Adjusted R-squared value followed by standard error and *P*-value (significant values in boldface).

**Species**	R^2 **± SE, *P* value**
Apis mellifera	0.027 ± 3.71, *P* = 0.111
Bombus terrestris	0.163 ± 0.65, ***P* =0.001**
Halictus scabiosae	-0.016 ± 0.86, *P* =0.789
Lasioglossum malachurum	0.019 ± 1.50, *P* =0.150
Xylocopa olivieri	0.009 ± 1.51, *P* =0.228
Xylocopa violacea	0.001 ± 0.10, *P* =0.306

Table S4. Results from ANCOVA testing effects of short-term heat exposure (4 hours at 38 °C) on CT_{Max} of six bee species, measured immediately after heat exposure and 24 hours later.

**Factor**	X^2	**DF**	***P*-value**
ITD	2.73	1	0.10
Species	28.97	3	**<0.001**
Treatment	0.01	1	0.93
Timing	0.01	1	0.91
Species×Treatment	3.57	3	0.31
Species×Timing	2.30	3	0.51
Treatment×Timing	0.69	1	0.41
Species×Treatment×Timing	1.85	3	0.60

Table S5. Critical thermal maxima (CT_{Max}) of bees following a 4-hour acute heat exposure (38 °C) and 24 hours after acute heat exposure (treatment groups). Control bees were kept at 27 °C and measured CT_{Max} at 4h and 24 h. Standard error (±) followed by sample size.

	**Control**		**Treatment**	
**Species**	**4 h**	**24 h**	**Immediately after 4 h of acute heat exposure**	**24 h after a 4-h heat exposure**
Apis mellifera	43.96±0.28, *n* = 20	44.58±0.35, *n* = 22	43.44±0.28, *n* = 20	44.64±0.26, *n* = 26
Bombus terrestris	42.61±0.33, *n* = 10	42.36±0.50, *n* = 11	42.58±0.48, *n* = 10	43.34±0.24, *n* = 12
Halictus scabiosae	43.29±0.44, *n* = 17	42.91±0.32, *n* = 24	45.03±0.19, *n* = 25	42.97±0.40, *n* = 19
Lasioglossum malachurum	42.23±0.15, *n* = 22	42.23±0.17, *n* = 20	42.09±0.13, *n* = 22	42.11±0.17, *n* = 20
